# Deep learning for prediction of post-thrombectomy outcomes based on admission CT angiography in large vessel occlusion stroke

**DOI:** 10.3389/frai.2024.1369702

**Published:** 2024-08-01

**Authors:** Jakob Sommer, Fiona Dierksen, Tal Zeevi, Anh Tuan Tran, Emily W. Avery, Adrian Mak, Ajay Malhotra, Charles C. Matouk, Guido J. Falcone, Victor Torres-Lopez, Sanjey Aneja, James Duncan, Lauren H. Sansing, Kevin N. Sheth, Seyedmehdi Payabvash

**Affiliations:** ^1^Section of Neuroradiology, Department of Radiology and Biomedical Imaging, Yale School of Medicine, New Haven, CT, United States; ^2^Institute of Clinical Pharmacology, University Hospital of RWTH Aachen, Aachen, Germany; ^3^Department of Biomedical Engineering, Yale School of Engineering, New Haven, CT, United States; ^4^Department of Radiology, University of California, San Diego, San Diego, CA, United States; ^5^CLAIM - Charité Lab for Artificial Intelligence in Medicine, Charité Universitätsmedizin Berlin, Berlin, Germany; ^6^Division of Neurovascular Surgery, Department of Neurosurgery, Yale University School of Medicine, New Haven, CT, United States; ^7^Division of Neurocritical Care and Emergency Neurology, Department of Neurology, Yale University School of Medicine, New Haven, CT, United States; ^8^Center for Brain and Mind Health, Yale University School of Medicine, New Haven, CT, United States; ^9^Department of Radiation Oncology, Yale School of Medicine, New Haven, CT, United States; ^10^Division of Stroke and Vascular Neurology, Department of Neurology, Yale University School of Medicine, New Haven, CT, United States

**Keywords:** deep learning, stroke, thrombectomy, CT angiography, outcome

## Abstract

**Purpose:**

Computed Tomography Angiography (CTA) is the first line of imaging in the diagnosis of Large Vessel Occlusion (LVO) strokes. We trained and independently validated end-to-end automated deep learning pipelines to predict 3-month outcomes after anterior circulation LVO thrombectomy based on admission CTAs.

**Methods:**

We split a dataset of 591 patients into training/cross-validation (n = 496) and independent test set (n = 95). We trained separate models for outcome prediction based on admission “CTA” images alone, “CTA + Treatment” (including time to thrombectomy and reperfusion success information), and “CTA + Treatment  + Clinical” (including admission age, sex, and NIH stroke scale). A binary (favorable) outcome was defined based on a 3-month modified Rankin Scale ≤ 2. The model was trained on our dataset based on the pre-trained ResNet-50 3D Convolutional Neural Network (“MedicalNet”) and included CTA preprocessing steps.

**Results:**

We generated an ensemble model from the 5-fold cross-validation, and tested it in the independent test cohort, with receiver operating characteristic area under the curve (AUC, 95% confidence interval) of 70 (0.59–0.81) for “CTA,” 0.79 (0.70–0.89) for “CTA + Treatment,” and 0.86 (0.79–0.94) for “CTA + Treatment + Clinical” input models. A “Treatment + Clinical” logistic regression model achieved an AUC of 0.86 (0.79–0.93).

**Conclusion:**

Our results show the feasibility of an end-to-end automated model to predict outcomes from admission and post-thrombectomy reperfusion success. Such a model can facilitate prognostication in telehealth transfer and when a thorough neurological exam is not feasible due to language barrier or pre-existing morbidities.

## 1 Introduction

In current stroke guidelines, advanced imaging techniques hold a crucial role in the decision-making process for treatment triage of Large Vessel Occlusion (LVO). Among these advanced imaging modalities, Computed Tomography Angiography (CTA) is a pivotal tool. It serves not only for evaluating treatment eligibility but also for assessment of arterial collateral supply and predicting functional stroke outcome prognosis. It has also been proven that CTA is more sensitive in detecting early infarction signs compared to non-contrast Computed Tomography (CT) (Camargo et al., 2007). Infarct cores determined on CTA images are strongly correlated with lesions defined on diffusion-weighted MRI (DWI) scans (Schramm et al., 2004). Recent studies have also revealed CTA’s potential to provide valuable information for long-term prognostication (van Seeters et al., 2015; Sallustio et al., 2017).

Recently, the emergence of artificial intelligence (AI) models has opened new possibilities for the prediction of long-term outcome from baseline stroke imaging information. These models enable the extraction of prognostic information directly from admission CTA scans, offering the potential to forecast patients’ outcomes. Unlike previous approaches that relied on manually engineered imaging biomarkers or complex preprocessing steps (Avery et al., 2022; Zhang et al., 2023), our approach streamlines the prediction process by taking the 3D images as input with minimal preprocessing in the end-to-end automated pipeline that uses raw unprocessed CTA scan to generate long-term outcome predictions. The disadvantage of complex preprocessing is the risk of information loss and the risk of introducing unknown biases. Deep learning makes it possible to keep the preprocessing steps small and at the same time preserve biomarkers that are currently unknown and not easily visually discernible.

In this study, we trained and tested separate deep learning models to predict 3-month outcomes after LVO thrombectomy from admission CTA scans with and without additional treatment and clinical variables. We compared models’ performance in independent test cohort and analyzed model biases. Such tools can facilitate objective prognostication of LVO stroke patients in acute setting. The prognostication of stroke outcomes by the presented models is especially useful for situations with the absence of reliable neurological exams and provides support for informed discussion regarding outcomes with patients and family members as well as establishing long-term goals of stroke management.

## 2 Methods

### 2.1 Study design

The clinical and imaging information for this study were retrieved from the stroke registry of Yale New Haven Hospital – from January 1, 2014, to October 31, 2020. Inclusion criteria were baseline CTA scan with at least 1-mm thickness axial slice available, anterior circulation LVO, attempted mechanical thrombectomy, and clinical outcome metrics. The 3-month follow-up modified Rankin Scale (mRS), or the closest interval to three months from stroke onset available was used to assess clinical outcome, and binarized to favorable (mRS ≤ 2) versus poor (mRS > 2). Exclusion criteria were suboptimal CTA scan quality due to motion degradation, metal artifacts, or scanner-related complications. We obtained the approvals from the institutional review board for the process of retrospective data collection. Informed consent was not sought from participants, as it was waived by the respective IRB. All procedures conducted during this study were adhered to current institutional and national guidelines.

### 2.2 Image preprocessing and training parameters

All head CTAs were resized to a common image dimension of 128x128x128 voxel using a template and resampled to a common voxel space of 1.5×1.5×1.5 mm using trilinear co-registration. The original images had a median (interquartile) voxel spacing of 0.47 (0.43–0.50) mm, 0.47 (0.43–0.50) mm, and 0.64 (0.63–0.63) mm, for the x-, y-, and z-axis, respectively. The template, resizing, and resampling was performed by applying the methodology described by (Sharrock et al., 2021) and (Rorden et al., 2012) using the Python implementation of the open-source medical package Advanced Neuroimaging Tools (Avants et al., 2011). No intensity scaling, image cropping or limit values of voxels have been used.

We leveraged a pretrained ResNet-50 3D Convolutional Neural Network (CNN) model named “MedicalNet” (Chen et al., 2019), initially trained on CT and MRI images for segmentation of multiple organs in the 3DSeg-8 dataset of the MedicalNet developers. We applied the “MedicalNet” weights to the Medical Open Network for AI (MONAI) ResNet-50 configuration (Cardoso et al., 2022) and performed a training process to fine-tune the pre-trained weights for binary classification in this study. For the training process, we partitioned the dataset with stratified splitting into a 5-folded training/cross-validation cohort (n = 496) and independent test (n = 95) and used a batch size of 6, maximum of 300 epochs, learning rate of 1×10^−6^, and a weight-decay regularization of 0 using Adam optimization.

To enhance the training process, we incorporated data augmentation techniques, encompassing rotations (with a 30% probability for each axis, within the range of −0.2 to 0.2 radians), zooms (with a 30% probability between 0.8 and 1.2 times zoom), flips (with a 20% probability), shear, and translations (with a 30% probability at 0.3). These augmented images were input into the ResNet model for each of the five cross-validation folds.

### 2.3 Training pipeline and model inputs – CTA images, “treatment,” and “clinical” variables

We trained, validated, and tested three separate joint models with inputs from (1) “CTA,” with only admission CTA scans as input; (2) “CTA + Treatment,” with input including admission CTA scans and “treatment” variables – i.e. admission-to-scan and scan-to-puncture time gaps and post-thrombectomy reperfusion efficacy. The post-thrombectomy reperfusion efficacy was determined based on a modified Thrombolysis in Cerebral Infarction (mTICI) system (Zaidat et al., 2013) as documented by neurointerventionalists. These values were converted into 0-to-4 ordinal variables. (3) “CTA + Treatment + Clinical,” with input including CTA, “Treatment,” and “Clinical” variables – i.e. admission NIH stroke scale (NIHSS), patient’s age, and sex.

For the training of our CTA-based model, we processed patient CTA images through a CNN using established preprocessing and training parameters. We selected the model with the lowest validation loss from each of the 5-fold cross-validation iterations, creating five distinct sub-models. These sub-models collectively determine the likelihood of a negative outcome by averaging their probability outputs. This approach mitigates the risk of overestimating the model’s performance on the test set. We then converted the final probability into a class prediction using a threshold optimized for accuracy on the validation set.

**Figure 1:**
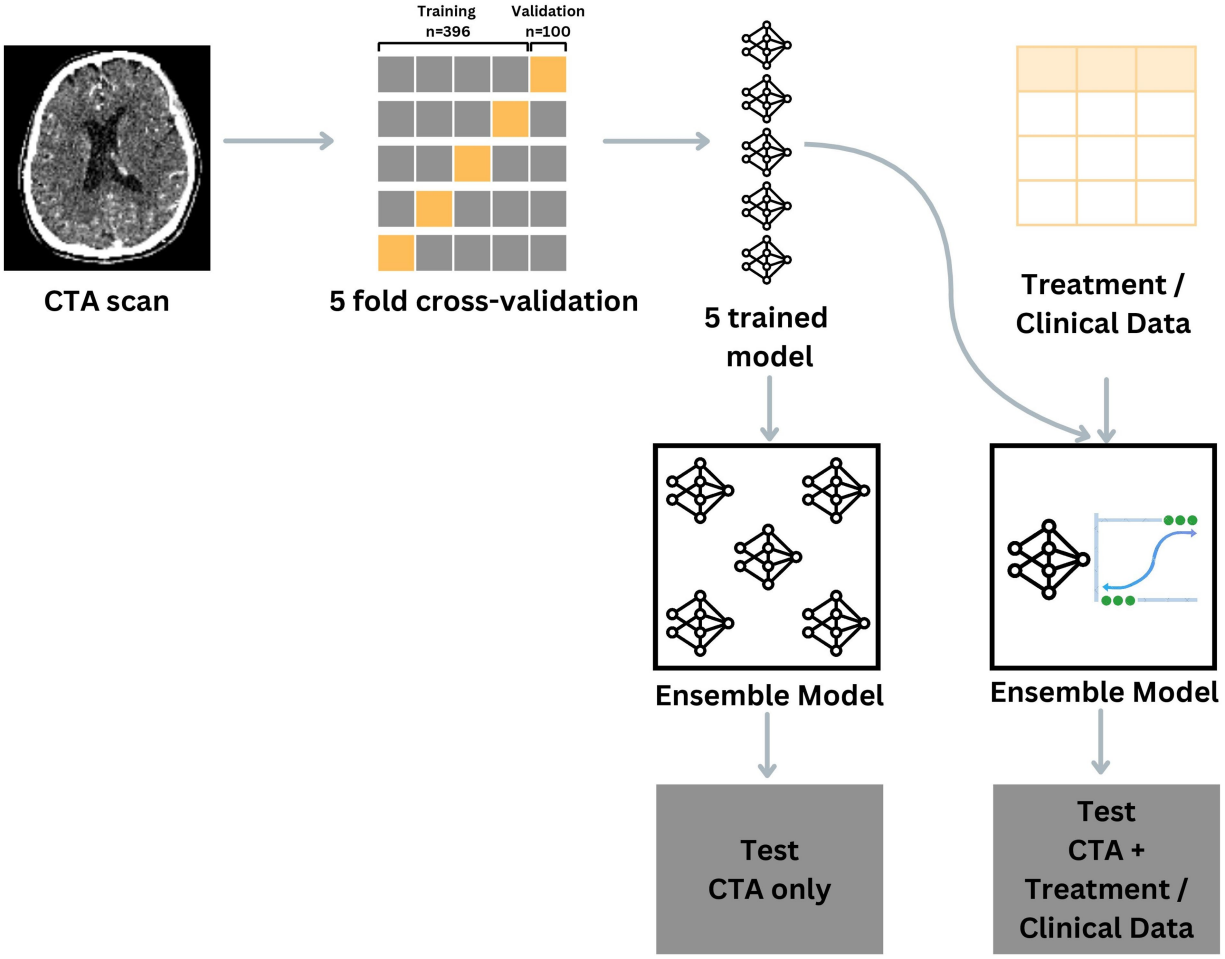
Visual description of the training, validation and testing process depending on the model input.

Subsequently, the five sub-models collectively predicted the probability of poor outcomes for each patient within the test set. The Area Under the Curve (AUC) of the Receiver Operating Characteristic (ROC) was then generated based on the mean probabilities per patient to assess the model performance. The whole process of the training and testing are depicted in Figure 1.

For the training of the ensemble model with multimodal input options, we used the probabilities for individual patients by passing the CTA images through the same models. Secondly, the probabilities are used alongside the other numerical input variables to train a logistic regression model using the validation set. The process is repeated for each cross-validation iteration, thereby creating 5 sub-models. The testing process is similar to the testing process of the “CTA” input pipeline, as the sub-models collectively predict the probability of poor outcomes for each patient within the test set. The optimal threshold to convert the probabilities into a class prediction is determined by the validation set in a similar manner as previously described.

In addition to deep learning models, we trained separate logistic regression models for prediction of outcome based on “Treatment” and “Treatment + Clinical” inputs on training/cross-validation cohort and test their performance in the independent test cohort. Since hyperparameter optimization was not needed when using a logistic regression model, we used the whole training/cross-validation cohort as input. Therefore, only one model instead was created based on “Treatment” or “Treatment + Clinical” inputs. We compared the performance of different models ROC AUC, using the DeLong et al. (1988) method.

### 2.4 Visual verifications of model attention

We applied the M3d-CAM (Gotkowski et al., 2020) to generate attention maps to visualize the regions in the input CTA scans that influenced the model prediction the most, deduced from the 4th layer of the model based on the first cross-validation set. These attention maps improve the interpretability of model predictions for human eyes and highlight head CTA regions with the highest impact on classification decisions by the deep learning model.

### 2.5 Model bias analysis

We organized the patients within the test set based on the predictions made by each model in comparison to the ground truth. Specifically, we grouped patients who were incorrectly predicted to have either a favorable or unfavorable outcome versus those who were accurately predicted to have either a favorable or unfavorable outcome.

### 2.6 Code and libraries

The analyses were conducted with Python 3.10.8, Visual Studio Code 1.72, R 4.3.1 and RStudio Server 2023.06.2. Some of the important Python modules being used are ANTs 0.3.8, Monai 1.1.0, PyTorch and PyTorch-Lightning 2.0.0, Scikit-learn 1.2.2 and M3d-CAM.

## 3 Results

### 3.1 Patients characteristics

A total of 591 patients were included in our analysis. The average age of patients was 70.2 ± 15.0 years, 322 (54%) were male, with median (interquartile) admission NIHSS of 14 (10–19), an average onset to CTA scan of 5.4 ± 5.5 h, and an average onset to catheterization of 7.1 ± 5.0 h. Table 1 summarizes the patients’ characteristics in training/cross (*n* = 496) and independent test (*n* = 95) cohorts. There was no significant difference between the clinical characteristics of these two cohorts.

**Table 1:** Patients’ characteristics in training/cross-validation versus independent test cohorts.

	Training/cross-validation (*n* = 496)	Independent test (*n* = 95)	*p* value
Age (years)	70.29 ± 15.02	69.85 ± 16.01	0.80
Male sex	269 (54%)	53 (56%)	0.14
Admission NIHSS (median, interquartile)	14 (9–19)	16 (11–20)	0.09
Onset-to-catheterization time (hours)	7.12 ± 5.04	7.20 ± 5.58	0.89
Onset-to-CTA scan (hours)	5.35 ± 5.35	5.36 ± 5.79	0.98
Occlusion side (left)	224 (55%)	40 (42%)	0.65
Internal carotid occlusion	110 (22%)	27 (28%)	0.25
Middle cerebral artery - M1 occlusion	299 (60%)	62 (65%)	0.66
Middle cerebral artery – proximal M2 occlusion	169 (34%)	22 (23%)	1.00
Received intravenous rt-PA	189 (38%)	36 (38%)	0.81
Post-thrombectomy reperfusion *	3 (3–3)	3 (1.50–3)	0.44
Post-thrombectomy hemorrhagic transformation	221 (45%)	51 (54%)	0.10
Missing 3-months mRS	124 (25%)	25 (26%)	0.79
Confirmed thrombolic events	131 (26%)	20 (21%)	0.33
Hypertension	270 (54%)	57 (60%)	0.38
Diabetes Mellitus	256 (52%)	50 (53%)	0.94

Ordinal values have been tested with the Chi2-Test, means have been compared with the Student’s *t*-test and median and interquartile ranges have been compared with the Mann–Whitney-*U* test. * A 0-to-4 ordinal variable was applied to represent post-thrombectomy reperfusion based on modified Thrombolysis in Cerebral Infarction (0, 1, 2a, 2b, and 3).

### 3.2 Model performance

**Figure 2:**
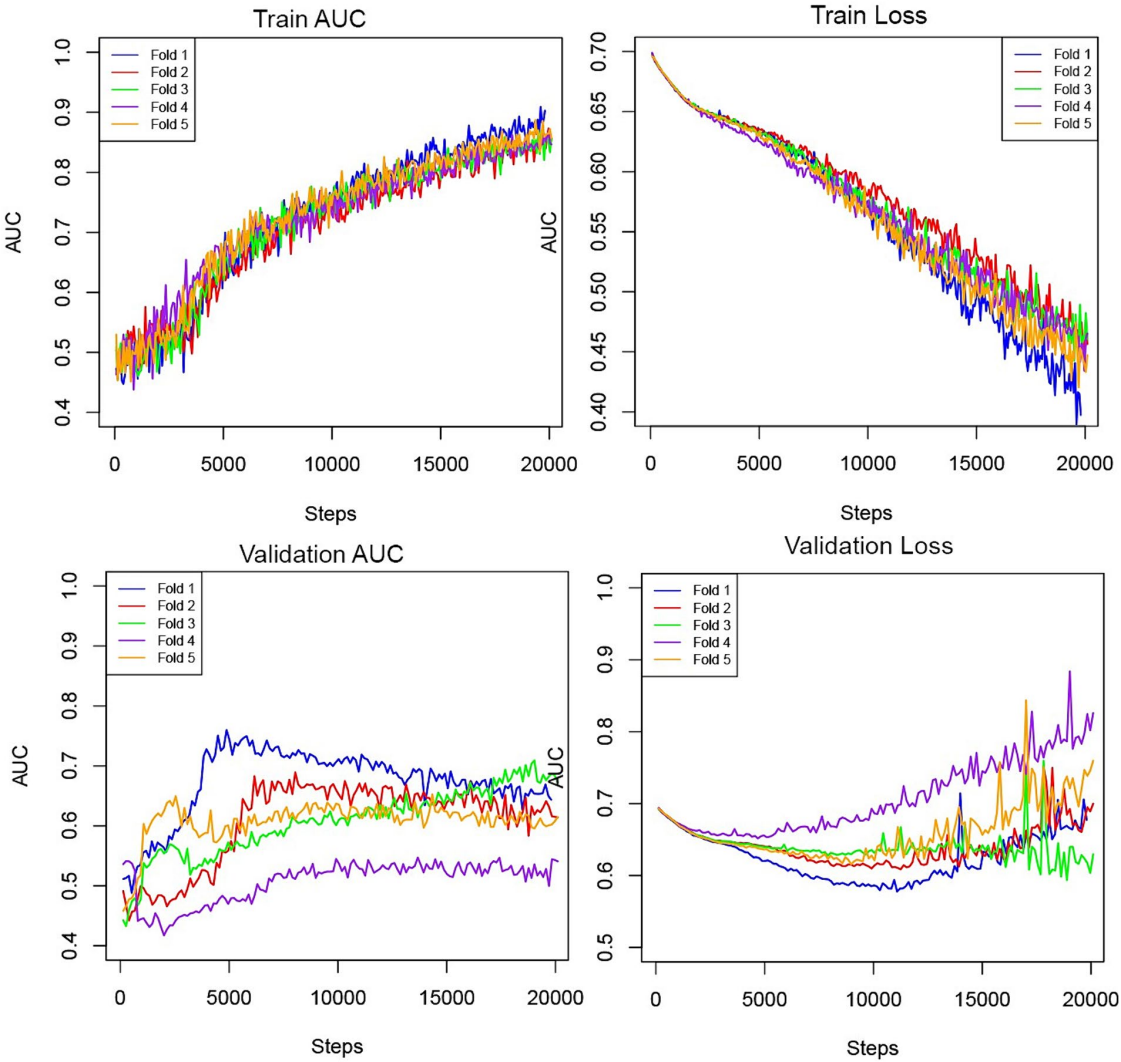
Training and validation AUC and losses throughout the steps in the five-fold cross-validation process.

**Figure 3:**
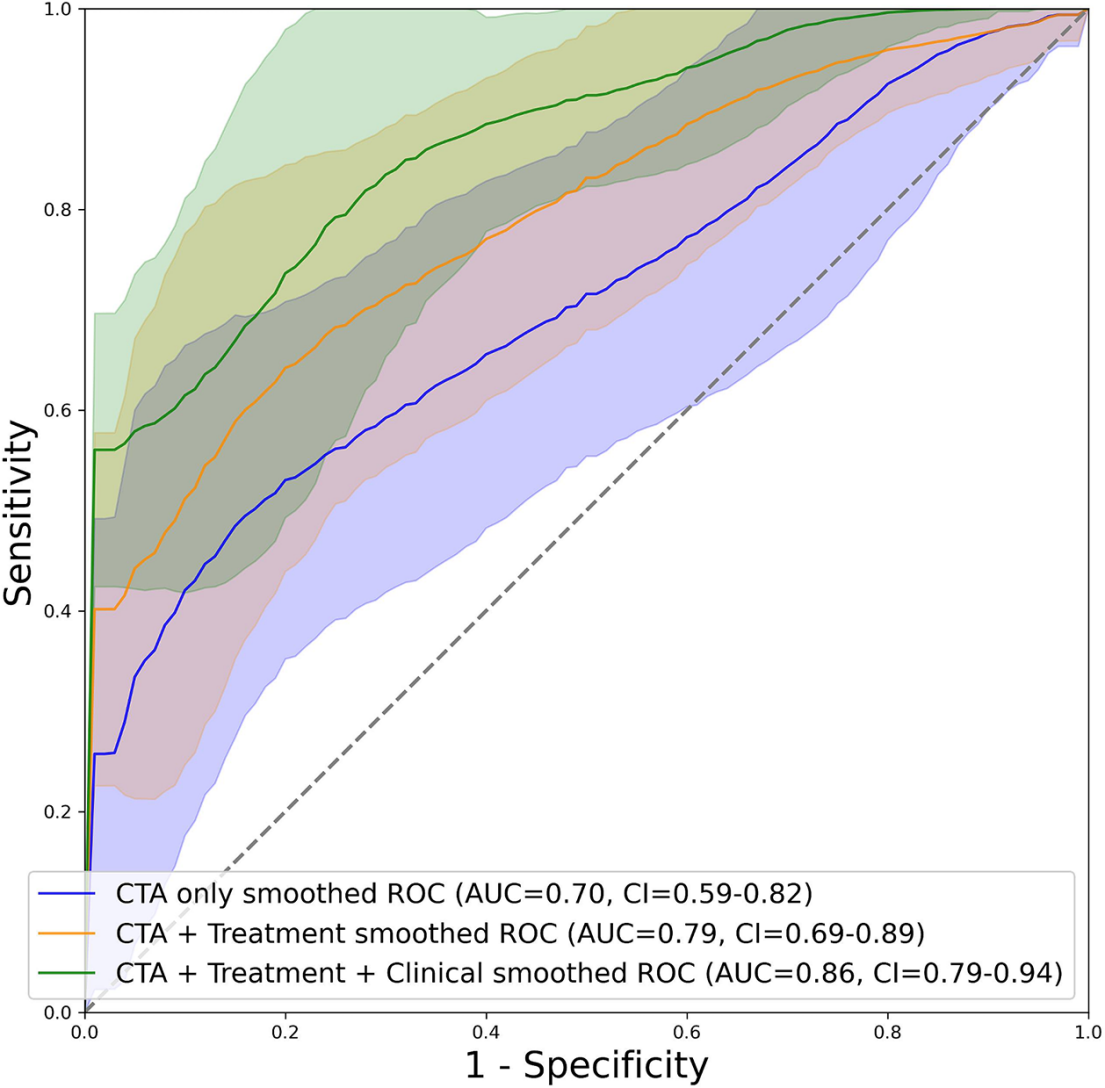
Graphical depiction of the AUC curve of mean probabilities of the “CTA”, “CTA + Treatment” and “CTA + Treatment + Clinical” model across all folds for the test set including their respective confidence interval.

**Figure 4:**
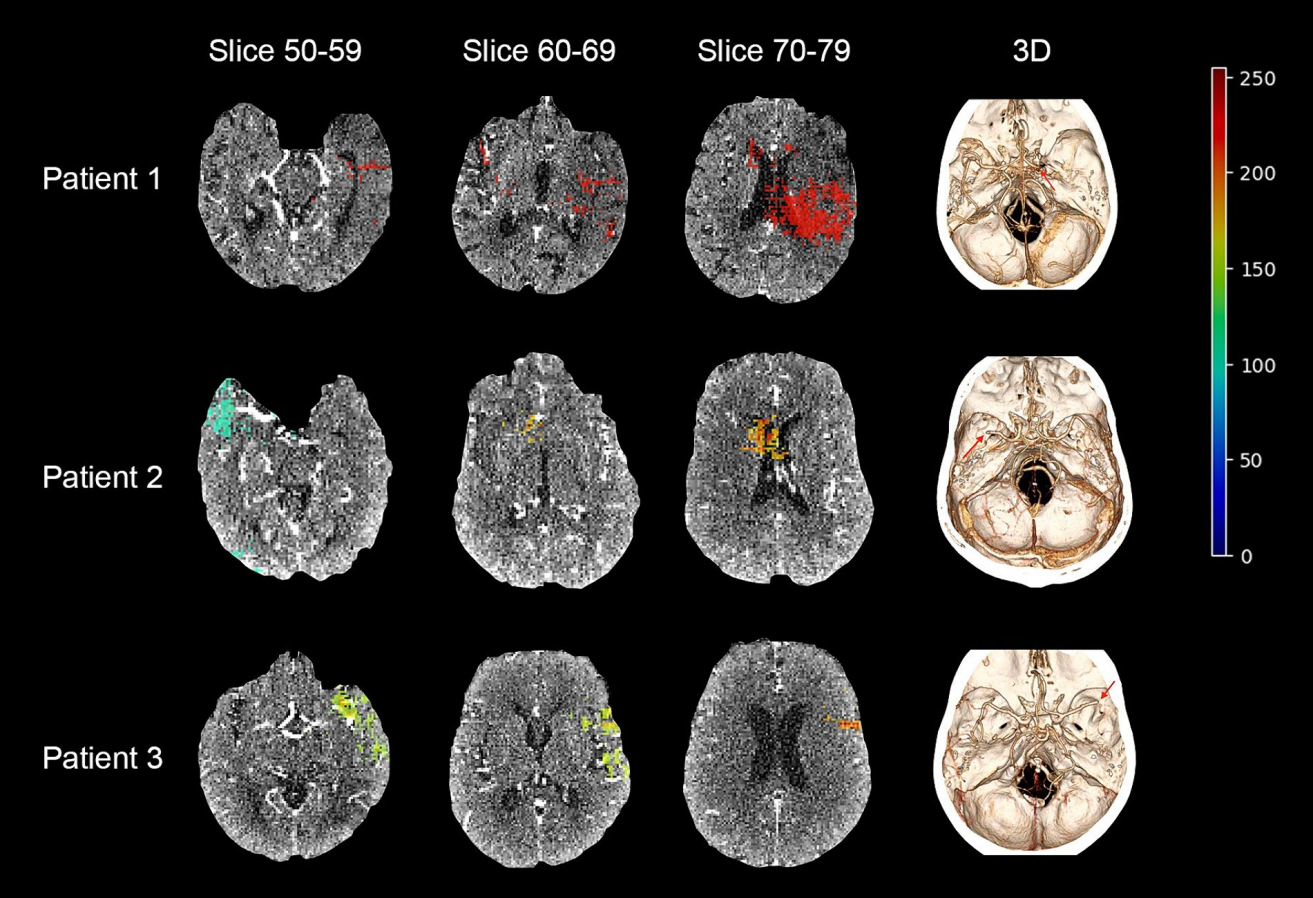
Maximum intensity projection (MIP) on a class activation map of three example patient in our cohort and a 3D visualization of each patient’s cerebral vessels. From each MIP only the upper half of the activation between the max and min activation are displayed. The area of occlusion is marked with a red arrow in the 3D visualization. Patient 1: probability for poor outcome = 0.67, label = 1; Patient 2: probability for poor outcome = 0.35, label = 0; Patient 3: probability for poor outcome = 0.78, label = 1.

Figure 2 depicts the AUC and loss function through 5-fold training and cross-validation. The final ensemble models with “CTA,” “CTA + Treatment,” “CTA + Treatment + Clinical,” “Treatment” and “Treatment + Clinical” achieved AUC (95% confidence interval) of 0.70 (0.59–0.81), 0.79 (0.70–0.89), 0.86 (0.79–0.94), 0.73 (0.61–0.85) and 0.86 (0.79–0.93) in independent test cohort, respectively. The AUC curves of the “CTA,” “CTA + Treatment” and “CTA + Treatment + Clinical” models are depicted in Figure 3. There was no significant difference between “CTA + Treatment” versus “Treatment” model AUC (*p* = 0.32), “CTA + Treatment” versus “Treatment + Clinical” model (*p* = 0.23), or “CTA + Treatment + Clinical” versus “treatment + clinical” model (*p* = 0.86). The attention maps projected over 15 mm thickness Maximum Intensity Projection slices of CTA scans reveal the area of brain with the highest attention across the input image (Figure 4).

### 3.3 Model bias analysis

Comparison of 74 patients with correct prediction versus 21 with incorrect prediction of outcome is summarized in Table 2. Overall, patients with incorrect prediction had better outcome (lower mRS), less severe baseline neurological symptoms (lower NIHSS), younger age, and higher post-thrombectomy reperfusion scores compared to those with correct prediction. This shows that the prediction model is biased toward overestimation of poor outcomes.

**Table 2:** Model bias analysis comparing the characteristics of patients with correct versus incorrect prediction by “CTA + Treatment” input model.

“CTA” model	True prediction (*n* = 73)	False prediction (*n* = 22)	*p*-value
3-month modified Rankin Scale	4 (4–6)	1.5 (1–2)	< 0.01
Sex (male)	41 (56%)	12 (55%)	1.00
Admission NIH Stroke Scale	17 (13–21)	11 (8–16)	< 0.01
Age (years)	71.97 ± 15.60	62.82 ± 15.67	0.02
Post-thrombectomy reperfusion *	0.25 (0–3)	3 (3–4)	< 0.01

“CTA + Treatment” model	True prediction (*n* = 74)	False prediction (*n* = 21)	*p*-value
3-month modified Rankin Scale	4 (4–6)	2 (1–2)	< 0.01
Sex (male)	43 (58%)	10 (47%)	0.90
Admission NIH Stroke Scale	17 (13.25–21.75)	11 (8–16)	< 0.01
Age (years)	72.03 ± 15.71	62.19 ± 14.99	< 0.01
Post-thrombectomy reperfusion *	3 (1–3)	3 (3–4)	0.03

*A 0-to-4 ordinal variable was applied to represent post-thrombectomy reperfusion based on modified Thrombolysis in Cerebral Infarction (0, 1, 2a, 2b, and 3).

## 4 Discussion

Our study demonstrates that by utilizing admission CTA scans, an automated model can accurately predict 3-month outcomes after thrombectomy in patients with LVO stroke. The end-to-end pipeline, which involves the preprocessing of admission CTAs, can be smoothly integrated into the clinical workflow. Furthermore, although not achieving statistical significance, we showed that the addition of post-thrombectomy reperfusion can increase the AUC of model predictions in the independent test cohort. The inclusion of clinical predictors such as age, sex, and neurological severity alongside CTA data did not significantly enhance model performance. Hence, CTA-based models can be especially useful in situations where clinical information is unreliable, such as in tele-stroke settings, language barriers, or when pre-existing morbidities make neurological exams challenging. It is worth noting that CTA scans are the *de facto* first line of imaging used to screen and diagnose LVO stroke, meaning that images are likely available before a complete clinical exam. Thus, a model that incorporates CTA data and accounts for reperfusion success can provide valuable prognostic information prior to the initial neurological exam.

Recent clinical trials (RESCUE–Japan LIMIT, SELECT-2, ANGEL-ASPECT, and TENSION) have demonstrated improved functional outcomes after thrombectomy, even among patients with a large infarct core and anterior circulation LVO (Yoshimura et al., 2022; Bendszus et al., 2023; Huo et al., 2023; Sarraj et al., 2023). In this context, the focus of stroke imaging workflow should shift toward identifying patients with LVO and thus eligible for thrombectomy (regardless of infarct core estimates). Subsequently, it should also identify patients at higher risk of poor outcomes despite thrombectomy – or clinically ineffective reperfusion. These patients are potential candidates for post-thrombectomy treatments, such as neuroprotective or neuroregenerative therapies. Deep learning models, such as the one developed and validated in this study, can provide this critical information.

However, other groups have also applied deep learning models for the prognostication of stroke. One research group applied a “Structured Receptive Field Neural Network” (RFNN), a variant of the ResNet model, with emphasis on the advantages of RFNN models in the context of smaller training datasets (Hilbert et al., 2019). They reported an average AUC of 0.71 for their best-performing model. Despite the complexity of their model and the presumable advantage in training with small datasets, their results did not exceed ours. Notably, their model generated a single 2D image from CTA scans for the prediction task. Their group also provided no comparison with models containing admission clinical information (Hilbert et al., 2019).

Another study employs a specialized model architecture called a “Siamese network” to focus on hemispheric asymmetries (Oliveira et al., 2023). This approach utilizes a more complex model structure, but it has the advantage that images of varying qualities could be assessed more uniformly. This model type utilizes Non-Contrast CT (NCCT) images as the main input. The CTA images were only used to predict the presence or absence of stroke to use as one of the clinical inputs. For this, they were compressed to a 2D image using MIP (Maximum Intensity Projection). In the highest-performing version of this model, the research group achieved an AUC of 0.74 in a test set size of 60 patients. Notably, the group included patients both with and without LVO, whereas patients with LVO are far more likely to have worse outcomes (Smith et al., 2006), and thus the model could have differentiated between those with and without LVO; in contrast, we only included patients with LVO, therefore a more clinically homogenous patient cohort.

Furthermore, in another study the researchers extracted radiomics features from middle cerebral artery (MCA) regions of CTA scans. These were utilized for the prediction of 3-month outcomes in patients with LVO stroke (Avery et al., 2022). Comparing models with different inputs, “Radiomics + Treatment,” and “Radiomics + Treatment + Clinical,” achieved AUCs of 0.68, 0.74, and 0.82 in independent test cohort, respectively (Avery et al., 2022).

Yet, one of the strengths of our research is that we created a fully end-to-end automated preprocessing and prediction pipeline for anterior LVO strokes. Compared to the previously mentioned works, our preprocessing pipeline includes fewer steps and yet achieves the same or better results. For example, we avoided skull removal techniques for CTA images due to artifact risk, we utilized a pre-trained network to improve classification, and we refrained from using MIP preprocessed images as input to minimize data loss. The advantage of this is that we can reduce the error potential and bias of our algorithm. The ensemble model structure, in which several individually trained versions come to a joint decision, has not yet been reported for this task. Not only it minimizes the risk of overfitting, but it also allows the flexible addition of other clinical variables, without any negative impact on the performance of the image analysis. In our analysis, we compared how the model with clinical variables performed in contrast to the model without clinical variables. Another novelty of our method is the inclusion of thrombectomy success as a prognostic input. This variable can provide the best versus worst-case scenario prediction for complete reperfusion versus thrombectomy failure. The “CTA + Treatment” model holds great potential for prognostication in acute stroke settings, as it can provide a wide range of outcome predictions, from the least to the most favorable treatment results based on the presumed lowest to highest mTICI reperfusion. This approach enables the model to estimate the probabilities of 3-month outcomes based on potential thrombectomy success.

It’s worth noting that the model can solely rely on CTA scans, too, which is almost always available at the time of LVO diagnosis. Therefore, a model based on imaging information can provide rapid and objective predictions regardless of local expertise and inter-examiner variabilities.

In our work, we also compared ensemble models using different variables with each other and were able to do a comprehensive assessment of different input strategies. For example, we wanted to simulate situations in which the determination of NIHSS is not possible for various reasons (e.g., tele-stroke setting, pre-existing motor deficit due to musculoskeletal degenerative disease, or language barrier). Although the combination of age, NIHSS, sex, and treatment results emerge as a strong prognostic model (Cummock et al., 2023; Oliveira et al., 2023), a fully automated model based on imaging input alone can be useful for immediate risk stratification as soon as CTA scan is completed.

The use of an ensemble model as described by us also has the advantage of increasing generalizability. Since multiple versions of a model make a decision together by averaging each one’s probability, it results in less fluctuation between each patient and is less prone to overfit. Since it is widely known that deep learning models trained on a specific set of imaging characteristics may struggle to generalize to external datasets with different imaging properties (Li et al., 2023), we also took additional measures to avoid overfitting in the training process. For example, we applied data augmentation techniques as well as L2-regularization, as they are known to increase the generalizability of a trained model (Sanford et al., 2020; Li et al., 2023). Notably, prognostic models based on automated analysis of admission CTA can offer generalizable treatment guidance information given the widespread availability of CTA scans, even in rural areas. This additional information is provided regardless of the presence expert reviewers, and without need for additional advanced imaging (such as perfusion), extra radiation or contrast administration. Risk-stratification of patients can identify potential candidates for additional post-thrombectomy neuroprotective or neuroregenerative therapies.

Attention maps help visually illustrate the deep learning model’s perception and provide insight into the decision-making process of machine learning models. As depicted in Figure 4, cerebral areas within the MCA supply territory had the highest impact on the decisions of our models. These findings confirm that model predictions were based on attention to at-risk cerebral tissue on CTA scans of LVO stroke patients (Waqas et al., 2020).

Our study has limitations. In model bias analysis, we found that false predictions of our model more commonly involved patients with favorable clinical characteristics (Table 2). The class imbalance within the dataset, particularly within the lower mRS categories, may have exerted an influence on the model’s performance during both the training and testing phases. In theory, there are some strategies that can be applied in the future to mitigate the issue of overestimating classes. For example, generative adversarial networks (GANs) are increasingly applied to generate synthetic images that oversample minority classes (Islam and Zhang, 2020), therefore increasing the ability of the model to reliably detect minority classes. Other approaches are aimed at optimizing the analysis of the generated probabilities like prediction uncertainty (Zou et al., 2023) or using a conditional probability for bias correction in prediction (Alexandari et al., 2020).

Also, retrospective datasets may suffer from biases inherent in the data collection process. These biases could be related to patient selection, imaging protocols, or institutional practices. Moreover, the mTICI score included in the dataset were not from core laboratory and therefore subject to inter-examiner variability. In addition, stroke management and outcomes can evolve due to advancements in medical treatments, changes in clinical guidelines, or improvements in healthcare practices. Retrospective studies without external validation might not account for these temporal changes, affecting the model’s applicability to more recent patient cohorts.

## 5 Conclusion

We showed the feasibility of an end-to-end fully automated to predict post-thrombectomy outcomes from readily available admission CTA images and treatment data. The model can be practically useful in the absence of a reliable neurological exam which can also provide robust prognostication. The comparison of our work to existing literature suggests that a simple deep learning approach, as implemented in our model, strikes a pragmatic balance between performance, architectural simplicity, and preprocessing ease.

## Data availability statement

The data analyzed in this study is subject to the following licenses/restrictions: unknown. The datasets analyzed during the current study are available from the corresponding author on reasonable request. The programming code used for analysis can be found at: https://github.com/Fledermaus12/LVOstroke-DL. Requests to access these datasets should be directed to SP, sam.payabvash@yale.edu.

## Author contributions

JS: Software, Writing – original draft, Writing – review & editing. FD: Formal analysis, Software, Writing – review & editing. TZ: Software, Writing – review & editing. AT: Software, Writing – review & editing. EA: Data curation, Writing – review & editing. AdM: Data curation, Writing – review & editing. AjM: Data curation, Writing – review & editing. CM: Data curation, Writing – review & editing. GF: Data curation, Writing – review & editing. VT-L: Data curation, Writing – review & editing. SA: Data curation, Software, Writing – review & editing. JD: Data curation, Writing – review & editing. LS: Data curation, Writing – review & editing. KS: Data curation, Writing – review & editing. SP: Conceptualization, Funding acquisition, Methodology, Project administration, Resources, Supervision, Writing – original draft, Writing – review & editing.
